# Comparative Analysis of Psychosocial Risks and Leadership in Municipal Government Agencies: A Study Based on NOM-035-STPS-2018 in Zacatecas, Mexico

**DOI:** 10.3390/ijerph23050639

**Published:** 2026-05-12

**Authors:** Sonia Villagrán Rueda, Lisandro José Alvarado-Peña, Luis Alfredo Vega Osuna, Flor de la Cruz Salaiza Lizárraga, Ma. Loecelia Guadalupe Ruvalcaba Sánchez, Bertha Lucía Santos-Hernández, Rodolfo Valentín Muñóz Castorena, Wilfred Fabián Rivera Martínez

**Affiliations:** 1 Academic Unit of Psychology, Universidad Autónoma de Zacatecas, Zacatecas 98066, Mexico; soniavillagran@uaz.edu.mx; 2 Academic Management, Universidad Tecnológica de Escuinapa, Escuinapa 82400, Mexico; 3 Economic-Administrative Department, Universidad Autónoma de Occidente, Culiacán Rosales 80020, Mexico; luis.vega@uadeo.mx; 4 Economic and Administrative Sciences, Instituto Tecnológico de Culiacán, Tecnológico Nacional de México, Ciudad de México 03330, Mexico; flor.sl@culiacan.tecnm.mx; 5 Centro de Investigación en Ciencias de Información Geoespacial, A.C., Ciudad de México 14240, Mexico; lruvalcaba@centrogeo.edu.mx; 6 Faculty of Administration, Accounting and Law, Universidad Autónoma de Coahuila, Coahuila 25000, Mexico; santosb@uadec.edu.mx; 7 Department of Quantitative Methods, Universidad de Guadalajara, Guadalajara 44100, Mexico; rodolfov@cucea.udg.mx; 8 Faculty of Administrative Sciences, Universidad Uniautónoma del Cauca, Popayán 190001, Colombia; wilfred.rivera.m@uniautonoma.edu.co

**Keywords:** psychosocial risks, leadership, work autonomy, public administration, occupational health

## Abstract

**Highlights:**

**Public health relevance—How does this work relate to a public health issue?**

- This study addresses occupational mental health in the Mexican public sector, identifying how command structure and the nature of service (operational vs. administrative) condition the psycho-affective well-being of workers. Specifically, departments such as municipal Public Safety present critical mental health risks due to high demand and low control.
- It utilizes the official standard NOM-035-STPS-2018 to evaluate psychosocial risks, providing empirical evidence on how lack of control and high work demands impact the health of public servants in vulnerable contexts.

**Public health significance—Why is this work of significance to public health?**

- The research reveals that critical departments such as Public Safety and Civil Protection present extreme risks due to workload and working hours, which correlates with the development of cardiovascular diseases and emotional exhaustion among workers.
- The study demonstrates that positive leadership acts as a protective factor that reduces the perception of mental workload, while the lack of recognition is a systemic problem that affects mental health and the sense of belonging throughout the municipal administration.

**Public health implications—What are the key implications or messages for practitioners, policy makers, and/or researchers in public health?**

- The findings suggest that policy makers must implement process reengineering and shift redesign, especially in operational sectors such as Public Safety, to mitigate the deterioration of mental health and prevent emotional exhaustion among staff.
- For practitioners and researchers, it is recommended to prioritize leadership training and the creation of non-monetary incentive programs as key strategies to strengthen the sense of belonging and ensure the operational sustainability of municipal institutions.

**Abstract:**

The public sector constitutes a complex system of psycho-affective management, where leadership and autonomy are crucial for institutional efficacy. The objective of this study was to analyze the relationship between leadership, autonomy (control over work), and other psychosocial risk factors across six departments of a government agency in Mexico, under the framework of NOM-035-STPS-2018. A quantitative, cross-sectional, and correlational design was utilized with 235 public servants, employing Reference Guide III of the official standard to identify psychosocial risk categories. The results, analyzed through Welch and Games–Howell tests, reveal significant differences based on the operational nature of each unit. Public Safety and Civil Protection present critical risks due to high demand and low control. The Municipal DIF (System for the Integral Development of the Family) stands out as a protective environment thanks to its positive leadership. A statistically significant association was identified between lower leadership quality and a decrease in work autonomy (*rho* = 0.701), along with a consistently low perception of performance recognition across all evaluated departments (*p* = 0.056). It is concluded that management style influences the perception of psychosocial risk, suggesting the need for interventions centered on humanistic leadership to mitigate occupational stress.

## 1. Introduction

Modern public administration is currently conceived as a complex system of uncertainty and continuous change, moving away from purely rational-bureaucratic structures. In this context, leadership style has been identified as a primary source of occupational stress, particularly in emergency scenarios [1]. Historically, stress research since the 1980s has demonstrated that institutional effectiveness depends not only on technical processes but on the management of Psychosocial Risk Factors (PRF). These are defined as aspects of work design, organization, and management that have the potential to cause physical, social, or psychological harm [2].

Within this ecosystem, leadership and control over work (autonomy) emerge as critical variables. International literature supports this relationship through the Demand-Control-Social Support model, where a worker’s decision-making capacity acts as a buffer against chronic stress [3]. Complementarily, ref. [4] Job Characteristics Model postulates that autonomy is an essential element for motivation and satisfaction; however, in contexts of high bureaucratic rigidity, normative structures often inhibit a leader’s capacity for innovation, neutralizing their relationship with employee well-being [5].

Similarly, ref. [6] research indicates that efficacy in modern institutions cannot be separated from the management of the psycho-affective processes of decision-makers, as emotional intelligence acts as a critical protective factor that reduces burnout and allows for sustained performance in high-pressure environments. This requires leadership capable of strengthening the emotional competencies of teams to guarantee the resilience of the administrative system in the face of uncertainty [7].

Nevertheless, the current state of research presents divergent hypotheses. While some authors suggest that positive leadership always fosters autonomy, other studies in highly rigid bureaucratic contexts maintain that inflexible normative structures often inhibit the leader’s innovative capacity, neutralizing their impact on well-being [5,8]. This tension is critical in operational roles, where the precariousness of autonomy limits professional development [9].

In the Mexican context, this issue is analyzed under the regulatory framework of NOM-035-STPS-2018. This standard identifies specific risk categories, such as negative leadership, lack of control over work, and excessive workload. The literature in ref. [10] notes that organizational structure in Mexico can act as either a risk or a protective factor depending on the command style. Furthermore, Ref. [11] highlights that empowerment through autonomy is an underutilized strategy to combat occupational stress. For their part, sectors such as public safety and municipal services present high risk factors due to the nature of their functions and the perceived lack of control [12,13].

At the regional level in Zacatecas, a gap in comparative research persists. Although isolated sectors have been explored [14], there has been no evaluation of how the nature of public service conditions leadership efficacy. Recent works suggest that autocratic styles predominate in security areas, limiting the perception of control [10,15]. Based on this premise, the present study seeks to address the following research question to what extent does the relationship between leadership style and the level of psychosocial risk vary according to the degree of autonomy across different municipal agencies in Zacatecas? We propose the following hypotheses: H1: There is a significant negative correlation between leadership quality and psychosocial risk levels. H2: The nature of the service (operational vs. administrative) moderates the relationship between leadership and psychosocial risk, with security agencies exhibiting a stronger association between deficient leadership and higher risk levels compared to administrative units, due to lower levels of perceived autonomy.

This study aims to contribute to the organizational literature by analyzing whether the influence of leadership on psycho-affective well-being is mediated by the rigidity of institutional protocols and the specific nature of departmental functions. Consequently, the general objective of this work is to analyze the relationship between leadership and psychosocial risk dimensions (specifically autonomy or control over work) in six government agencies in Zacatecas: Municipal Presidency, Municipal Public Safety, Civil Protection, House of Culture, National System for Integral Family Development (DIF), and the Zacatecas Municipal Institute for Women’s Equality (INMUZAI).

## 2. Materials and Methods

The present research was conducted through a quantitative, correlational, and comparative study, utilizing a non-experimental, cross-sectional design [16]. The study focused on analyzing the relationship between leadership, autonomy, and psycho-affective processes within a municipal government framework.

Setting and Selection of Departments. The six departments were selected through non-probabilistic convenience sampling. While the municipal government includes numerous other administrative units, these specific six were chosen because they effectively represent the entire operational spectrum of public service: from high-intensity, reactive, and high-risk operational roles (Public Safety, Civil Protection) to administrative and social service functions (Presidency, DIF, INMUZAI, House of Culture). This approach ensures that the selection provides the necessary diversity to enable a robust comparative analysis of psychosocial risk factors, capturing both the extreme ends of the institutional risk profile and the standard administrative conditions.

The selected departments encompass diverse job descriptions and operational conditions: Municipal Public Security and Civil Protection involve personnel (police officers and paramedics) characterized by high-intensity shifts, exposure to traumatic events, and a vertical command structure with low individual autonomy. In contrast, the Municipal Presidency and the Zacatecas Municipal Institute for Women’s Equality (INMUZAI) consist of administrative and technical staff focused on public policy, legal affairs, and gender-based human rights, where tasks are predominantly office-based. Finally, the DIF Municipal and the House of Culture include social workers, psychologists, and cultural promoters who manage high emotional labor due to their direct interaction with vulnerable populations and community engagement.

Participants and Sampling. A total of 310 public servants were invited to participate in the study, representing the total active workforce of the six selected departments during the evaluation period. From the initial invitations, 255 surveys were returned; however, 20 were discarded due to incomplete data or failure to meet the inclusion criteria, resulting in a final sample of 235 participants (*N* = 235) and a response rate of 75.8%. This sample size provides a 95% confidence level and a 5.8% margin of error for the specific population of these departments. Inclusion criteria required participants to have a minimum of six months of seniority to ensure a consolidated perception of the organizational climate and to be active employees under a formal contract at the time of the study.

Instruments and Variables. The diagnostic framework employed was Reference Guide III of the NOM-035-STPS-2018, an instrument validated by the Mexican Ministry of Labor and Social Welfare for workplaces with over 50 employees [2]. This instrument was selected because it allows for a standardized evaluation of psychosocial risk factors (PRF) in the Mexican context. Although Reference Guide III of the NOM-035-STPS-2018 is composed of five domains and several categories (totaling ten factors), this study focused specifically on five key dimensions (Work Environment, Activity-related Factors, Time Management, Leadership/Relationships, and Organizational Environment) to align directly with the study’s focus on demand-control and job characteristics models. The questionnaire consists of 72 items on a five-point Likert scale (0–4), where higher scores indicate higher levels of psychosocial risk.

Statistical Analysis and Hypothesis Testing. Data were processed using IBM SPSS Statistics (version 26; IBM Corp., Armonk, NY, USA). To test the research hypothesis (H1), which posits a negative correlation between leadership quality and psychosocial risk, Spearman’s rank correlation coefficient (*rho*) was utilized, as it is appropriate for non-parametric data and ordinal scales. To address the comparative objective between departments, a One-Way ANOVA was initially considered. However, since Levene’s test indicated a violation of the assumption of homogeneity of variances (*p* < 0.05) in dimensions such as Workload and Autonomy, the Welch’s Test was reported instead for its robustness in heteroscedastic conditions. Subsequently, the Games–Howell post hoc test was applied to identify specific significant differences between pairs of departments, particularly to contrast the high-risk operational areas (Security and Civil Protection) against administrative and social units.

## 3. Results

This section presents the findings derived from the analysis of psychosocial risk factors in the Municipal Government of the State of Zacatecas, following a logical structure that moves from the characterization of the sample to the specificities of its operational units. Initially, the sociodemographic properties of the participants are detailed, providing an essential comparative basis to contextualize the subsequent results. The organizational diagnosis begins with a visual approach using radar charts, which allow for a descriptive identification of the “risk area” and critical domains across the six evaluated departments. This perspective is further deepened through the application of a one-way ANOVA, the results of which reveal significant differences in risk perception according to the nature of the administrative unit. These disparities are further specified using the Games–Howell post hoc test by contrasting operational departments—such as Public Safety and Civil Protection—with administrative management areas. Finally, the association between variables is examined through a Spearman correlation matrix, analyzing how leadership and time organization relate to the autonomy and psycho-affective processes of the officials, avoiding the establishment of causal links and limiting the interpretation to the observed statistical interdependence between these factors.

As a starting point for the analysis, Table 1 details the sociodemographic profile of the participants, segmented by the primary comparison groups. This characterization not only allows for an understanding of the sample composition in terms of age, gender, tenure, and hierarchical level, but also establishes the necessary framework to rigorously interpret the variations in psychosocial risk perception presented in subsequent analyses. The distribution shown here guarantees the representativeness of the various operational and administrative units, ensuring the validity of the statistical contrasts performed.

The sociodemographic composition of the sample shown in Table 1 reveals a balanced distribution in terms of gender, with 51.5% female participation compared to 48.5% male, ensuring the representativeness of both groups in the perception of the work environment. Regarding age structure, a mature and productive workforce predominates; 46.0% of the officials fall within the 30 to 44-year range, followed by 32.3% in the 45 to 59-year group, suggesting significant generational stability within the local government. This stability is further reinforced by marital status, as the majority of employees (69.4%) reported being married or in a common-law union.

With respect to educational attainment, the sample is characterized by a notable level of professionalization: 43.4% of the public servants hold a bachelor’s degree and 9.8% have postgraduate training, accounting for more than half of the personnel with higher education. Nevertheless, there is a significant segment (31.5%) with upper secondary or technical education, which is relevant for contextualizing variations in the cognitive demands of the positions.

Finally, the job profile shows a predominantly operational and administrative structure (80.0%), which is consistent with the nature of municipal services. In terms of employment status, 60.4% hold a permanent (tenured) position, while 39.6% are under temporary or contract regimes. This contractual and hierarchical configuration is fundamental for the subsequent analysis of psychosocial risk factors, specifically in dimensions such as job security, autonomy, and the perception of leadership within the organization.

The sociodemographic baseline established above provides the necessary context to understand how individual backgrounds converge with the organizational environment. Nevertheless, to fully grasp the distribution of psychosocial stressors, it is essential to transition from an aggregate view of the workforce toward a localized examination of the various administrative units. Consequently, the following section is presented:

### 3.1. Comparative Analysis of Psychosocial Risk Profiles by Dependency

Analysis of the Range (The Vulnerability Factor)

**Figure 1:**
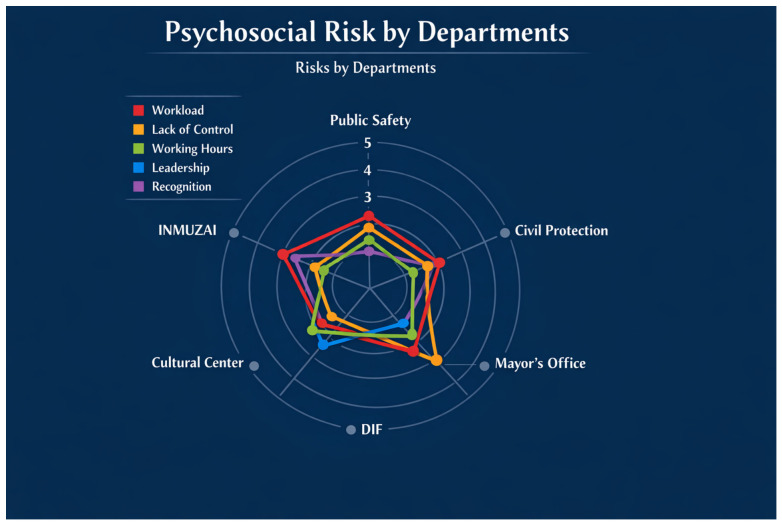
Comparative profile of psychosocial risks by government agency. Scores represent the standardized means of the NOM-035 domains. A critical expansion is observed in the Workload and Working Hours domains for operational personnel.

- Public Safety and Civil Protection (External Lines): The high vulnerability of these populations is evident (see Figure 1) where the “Workload” and “Working Hours” vertices reach their maximum limits, confirming a failure in the regulation of operational times.
- DIF (National System for Integral Family Development) and House of Culture (Internal Lines): More contracted areas closer to the center are observed. As illustrated in Figure 1, This indicates that, despite belonging to the same municipal administration, these agencies manage to maintain healthier psycho-affective processes, probably due to the nature of their tasks with lower emergency demands.

Vertex Analysis (Specific Domains)

- The “Leadership” Vertex: The Civil Protection point stands out even more than the Public Safety point on this axis (Figure 1). This suggests that the risk within that unit is not solely operational (e.g., firefighting or rescue) but is also significantly related to command and management structures.
- The “Lack of Control” Vertex: This axis is often “pulled” or stretched outwards in the same departments that have a high risk in Leadership. This visually confirms Spearman’s correlation (*rho* = 0.701); the graph (Figure 1) shows that where the leader is perceived negatively, the worker feels they lose control of their tasks.

The Convergence Phenomenon (Common Points)

- Performance Recognition: On this axis, all lines are at a similar distance from the center.
- Interpretation: This indicates that the feeling of “lack of appreciation” is a systemic cultural problem within the City Council that does not discriminate between police officers, administrative staff, or social workers.

### 3.2. Comparative Analysis of Interdependencies

Following the visual characterization of the risk profiles, a one-way ANOVA was performed to determine whether the operational nature of each department is associated with significant differences in the perception of psychosocial domains (see Table 2). This statistical analysis validates whether the institutional environment of each administrative unit relates to differentiated risk experiences for public servants, laying the groundwork for subsequent multiple comparisons.

The ANOVA results reveal highly significant differences (*p* < 0.001) in four of the five domains evaluated: Workload (F = 31.611), Working Hours (F = 20.148), Lack of Control (F = 13.271), and Leadership (F = 5.364). These values robustly indicate that psychosocial risk in the Municipal Council is not homogeneous; the administrative location of the worker is a factor that coincides with varying levels of exposure to these factors.

The results of the analysis of variance by domains are presented in Table 3. The Workload domain stands out, presenting the highest *F*-value, which suggests it is the dimension where departments (such as Public Safety compared to administrative areas) show the most profound discrepancies. Conversely, the Performance Recognition domain (*p* = 0.056) did not reach the threshold of statistical significance (*p* < 0.05). This finding suggests that the perception of low appreciation is a transversal phenomenon throughout the City Hall. Regardless of the unit, the perception of recognition remains constant, indicating a generalized characteristic in the institution’s organizational culture.

The findings presented in the analysis of variance and the breakdown by domains confirm that the Zacatecas City Council’s work ecosystem exhibits fragmented psychosocial risks. While a lack of recognition is a constant within the institution, critical factors such as workload and working hours depend directly on staff assignment. However, to accurately determine which specific departments exhibit significant differences and to ensure the validity of these comparisons against a heterogeneous sample, it is imperative to move toward a multiple contrast analysis.

### 3.3. Multiple Contrast Analysis: Post Hoc Test

Once the existence of significant overall differences was confirmed using ANOVA, the next technical step was to identify the pairs of dependencies where these discrepancies resided. For this purpose, the Games–Howell post hoc test was selected. The choice of this specific test was based on criteria of statistical rigor given the nature of the sample. Prior to the analysis, Levene’s test was performed, which yielded a significance level of less than 0.05, indicating that the principle of homogeneity of variances (heteroscedasticity) was not met.

Since the size of the groups per dependency is unequal and the variances are not constant, the Games–Howell test is the most robust and precise procedure, as it does not assume equal variances and maintains strict control over Type I errors in multiple comparisons. The results of the significant contrasts, which allow for the geographical identification of critical intervention points within the municipal structure, are presented in Table 4.

The multiple comparisons table reveals a critical gap between operational and administrative/social departments. The analysis yields three key findings:

1. The Critical Prevalence of Public Safety Issues

The Public Safety department stands out as the area of greatest risk in all evaluated domains. The mean differences are especially alarming in Workload (+17.32) and Lack of Control (+11.41) when compared to the Mayor’s Office and the DIF (National System for Integral Family Development). These values, with a significance level < 0.001, confirm that security personnel not only have more tasks but also possess the least autonomy to manage them within the municipal structure.

2. The DIF as a Benchmark in the Operational-Social Gap

The DIF is positioned as the department with the lowest risk levels, serving as the lowest point of contrast (J). The difference in averages for Leadership (+6.87) and Working Hours (+3.18) compared to Public Safety suggests that, while the DIF (National System for Integral Family Development) maintains more stable psycho-affective processes, the operational areas suffer significant deregulation in command and rest periods.

3. Shared Risks in Civil Protection and INMUZAI (Municipal Institute of Municipalities and Indigenous Organizations)

Civil Protection shows a significant difference in Workload (+13.70) and, notably, the highest disparity in Leadership (+7.54) when compared to the DIF. This suggests that, in emergency scenarios, leadership style is perceived as a major stress factor. INMUZAI, for its part, although a smaller agency, presents a significantly higher workload than the Presidency (+11.29), indicating operational saturation in its specific functions.

The differences observed in the Games–Howell contrasts allow for the geolocation of psychosocial risk within the municipal environment, evidencing a polarization where the greatest deficiencies in Management and Autonomy are concentrated in Public Safety and Civil Protection. These statistically significant mean disparities (*p* < 0.05) suggest that the nature of the public service provided is associated with specific variations in the staff’s psycho-affective processes.

However, since this methodological design does not allow for the establishment of cause-and-effect relationships, the following section is limited to examining the strength of association and the interdependence between these variables. To this end, Spearman’s rank correlation coefficient is used (see Table 5), which identifies how the perception of leadership covaries with control over work, describing the statistical relationship between both domains without assuming a causal direction.

### 3.4. Spearman’s Correlation

To conclude the results report, the strength of association between domains was analyzed using Spearman’s rank correlation coefficient *(rho*). This analysis identifies how institutional management (Leadership) is linked to the worker’s operational and psycho-affective experience (Autonomy and Recognition). The correlation matrix reveals significant associations across all evaluated dimensions (*p* < 0.001), highlighting three critical links for understanding job well-being within the City Council:

Leadership and Lack of Control *(rho* = 0.701). This is the most robust correlation in the study, indicating a strong positive association. The data suggest that the perception of deficient or authoritarian leadership coincides with a higher perception of lack of autonomy. In management terms, leadership and control over work exhibit a close interdependence.

Working Hours and Workload *(rho* = 0.715). Task saturation is positively correlated with the length of working hours. This finding is consistent with the profile observed in the Public Safety and Civil Protection areas, where high operational demands are associated with a deregulation of rest periods. Lack of Control and Recognition (*rho* = 0.584). There is a moderate-to-strong correlation linking autonomy with performance evaluation. The data suggest that personnel with less decision-making power over their processes tend to perceive themselves, simultaneously, as less recognized by the institution.

The statistical evidence presented confirms that job satisfaction in the public sector of the municipality in question is closely related to a command structure linked to limited operational autonomy. While the ANOVA analysis and post hoc tests identified Public Safety as the epicenter of psychosocial risk, the correlation matrix demonstrates that Leadership exhibits a systematic association with both the lack of control and the lack of recognition across all evaluated departments.

## 4. Discussion

The findings of this study allow for a contrast of the hypotheses regarding the heterogeneity of psychosocial risk within the public sector of Zacatecas. The results confirm that workplace well-being is not merely a product of individual resilience but is intrinsically associated with functional clarity and the regulatory capacity of the organizational structure. From the perspective of the Demand-Control-Support Model by ref. [3], the significant disparity detected between Public Safety and the Municipal DIF supports the classic theory that high psychological demands, when they coexist with low decision latitude (control), are related to higher levels of job strain. In the case of security officers, the rigid nature of their duties—dictated by emergency protocols—limits the “work autonomy” that acts as a critical stress buffer in more flexible organizational models.

In the context of public health in Mexico, these findings transcend administrative management. The prevalence of a “high-strain” scheme in operational sectors such as Public Safety and Civil Protection aligns with the work of ref. [17], who argues that employee burnout is not an isolated event but an epidemiological vulnerability that impacts the national healthcare system (IMSS, ISSSTE). Chronic exposure to these risks is linked to an increase in non-communicable diseases and mental disorders such as burnout. Within this framework, NOM-035-STPS-2018 must be reinterpreted as a strategic primary prevention tool rather than a mere bureaucratic requirement.

A contrasting and enriching finding emerges when analyzing the Municipal DIF, where the perception of stronger leadership and social support reinforces the “support” dimension of the Karasek model Ref. [18]. This coincides with the views of ref. [6], who argue that emotional intelligence in management can neutralize mental workload. It is here that the role of middle management as organizational “filters” becomes relevant. This concept refers to the ability of middle managers to act as a regulatory membrane: in a negative scenario, they can aggravate the effects of toxic top management by replicating authoritarian styles; however, in a positive scenario, they function as a “buffer” that protects operational staff from structural and budgetary pressures, translating top management demands into manageable instructions and emotional support.

Nonetheless, a cross-cutting result requiring attention is the uniform lack of performance recognition across all areas (*p* = 0.056). This trend suggests a systemic “culture of silence” regarding merit. According to the ref. [2], the absence of feedback is one of the most corrosive factors in the Mexican public sector, as it dissolves professional identity and fosters “institutional detachment.” Furthermore, the robust association identified between deficient leadership and lack of control (*rho* = 0.701) suggests that in traditional bureaucracies, the command style conditions the worker’s space for autonomy.

Finally, although this study offers a “snapshot” of the current state of the Municipal Government of Zacatecas, its cross-sectional nature limits the ability to establish definitive causality, thus adhering to a strictly associative interpretation. The data suggest that for the Mexican public sector to be truly efficient, it must ensure that its “human infrastructure” is protected from the very risks it is commissioned to mitigate in society.

### Study Limitations

Despite the relevance of the findings, this research has intrinsic limitations that must be considered when interpreting the results. First, the cross-sectional nature of the design prevents the establishment of definitive causal relationships between the variables studied; the data offer a “snapshot” of risk perception at a specific point in time, but do not capture the dynamic evolution of psychosocial factors over time. In this sense, future longitudinal studies would be necessary to confirm whether changes in leadership style produce direct variations in chronic stress levels.

Second, the use of self-report instruments (Reference Guide III of NOM-035-STPS-2018) carries the risk of social desirability bias. This factor is particularly critical in high-rigidity hierarchical environments such as Public Safety and Civil Protection, where workers might underestimate their risk levels due to fear of institutional reprisals or the cultural normalization of operational stress. Furthermore, the non-probabilistic purposive sampling, while allowing for the representation of contrasting operational realities, limits the generalizability of the results to other municipalities with different administrative structures or sociodemographic contexts.

Finally, it is important to acknowledge that the study focused exclusively on the dimensions covered by the Mexican regulation. External factors, such as the regional socioeconomic environment of Zacatecas or the personal living conditions of the employees, were not integrated into the correlational analysis, despite their potential influence on the mental health of public servants. Therefore, it is recommended that subsequent research employ mixed methods to delve deeper into the subjective meanings of autonomy and leadership in the public sector.

## 5. Conclusions

The present research allows for the conclusion that exposure to psychosocial risk factors within the Municipal Government of Zacatecas is not a uniform phenomenon; rather, it is intrinsically contingent upon the operational role and the administrative support structure of each unit. The findings confirm a marked sectoral vulnerability, particularly in departments such as Public Safety and Civil Protection. In these areas, the convergence of high psychological demands and low decision-making latitude creates a “high strain” environment which, according to ref. [3], constitutes the primary precursor to risks regarding the physical and mental health of public servants. This evidence underscores that the nature of the service provided is the determining factor in the disparity of risk perception.

Furthermore, it is concluded that positive leadership and social support act as critical protective resources, as observed in the management of the Municipal DIF. This contrast identifies that the command style of middle management functions as an axis that can either buffer or enhance occupational stress. Nevertheless, the study reveals a systemic institutional weakness: the transversal absence of recognition and feedback systems. This void not only contravenes the provisions of NOM-035-STPS-2018 regarding the sense of belonging but is also identified as a trigger for institutional detachment and burnout, regardless of hierarchical level or assigned department.

Ultimately, while the implementation of current regulations in the municipality appears to meet formal requirements, the data demonstrate that a significant gap remains in its practical application. For public service to be sustainable, occupational health must cease to be a bureaucratic compliance and instead become the foundation of organizational effectiveness. Ignoring these disparities not only erodes human capital but also compromises the operational capacity of the agencies most vital to Zacatecan society. In light of these primary findings, and with the aim of moving toward a genuine culture of prevention, the strategic recommendations detailed in Table 6 are proposed.

## Figures and Tables

**Table 1:** Sociodemographic characteristics of the participants (*N* = 235).

Variable	Category	Frequency (*n*)	Percentage (%)
Gender	Male	114	48.5%
	Female	121	51.5%
Age Group	15–29 years	42	17.9%
	30–44 years	108	46.0%
	45–59 years	76	32.3%
	60+ years	9	3.8%
Marital Status	Married/Common-law	163	69.4%
	Single	51	21.7%
	Divorced/Separated/Widowed	21	8.9%
Education Level	Basic (Primary/Secondary)	36	15.3%
	Intermediate (High School/Technical)	74	31.5%
	Higher Education (Bachelor’s)	102	43.4%
	Postgraduate (Master’s/PhD)	23	9.8%
Type of Position	Operational/Administrative	188	80.0%
	Professional/Technical	32	13.6%
	Managerial/Supervisor	15	6.4%
Contract Type	Permanent (Base)	142	60.4%
	Temporary/Contract	93	39.6%

**Table 2:** Comparative ANOVA analysis.

Variable (Domain)	Df	*F*	Sig. (*p*)
Workload	5.228	31.611	<0.001
Lack of Control	5.228	13.271	<0.001
Working Hours	5.228	20.148	<0.001
Leadership	5.227	5.364	<0.001
Recognition	5.227	2.188	0.056

**Table 3:** Breakdown by domains.

Domain	Result Interpretation	Organizational Impact
Workload	It is the domain with the highest F-value (31.611).	Indicates that task volume and accelerated work rhythms vary drastically between offices.
Lack of Control	Significant difference (F = 13.271).	Autonomy to take breaks or decide the speed of work depends entirely on the assigned agency.
Working Hours	Significant difference (F = 20.148).	Excessive hours and working on rest days are critical problems only in specific areas.
Leadership	Significant difference (F = 5.364).	The quality of the supervisor-subordinate relationship and clarity of roles vary according to the director of each area.

**Table 4:** Significant findings (Critical Differences).

Domain (Risk Factor)	Agency with HIGHER Risk (I)	Compared to (J)	Mean Difference (I–J)	Significance (*p*)
Workload	Public Safety	President’s Office	+17.32	<0.001 (Very high)
	Public Safety	DIF (Family Services)	+14.54	<0.001
	Civil Protection	President’s Office	+13.70	<0.001
	INMUZAI	President’s Office	+11.29	0.024
Lack of Control	Public Safety	DIF (Family Services)	+11.41	<0.001
	Public Safety	President’s Office	+9.17	<0.001
	Public Safety	INMUZAI	+8.54	0.009
Working Hours	Public Safety	DIF (Family Services)	+3.18	<0.001
	Public Safety	President’s Office	+3.15	<0.001
Leadership	Public Safety	DIF (Family Services)	+6.87	0.001
	Civil Protection	DIF (Family Services)	+7.54	0.039
Recognition	Public Safety	DIF (Family Services)	+3.27	0.024

**Table 5:** Spearman’s correlation matrix between psychosocial domains.

	Workload Domain	Lack of Control Domain	Working Hours Domain	Leadership Domain	Performance Recognition Domain
Workload Domain	-------				
Lack of Control Domain	0.429 **	--------			
Working Hours Domain	0.715 **	0.397 **	------		
Leadership Domain	0.294 **	0.701 **	0.266 **	-------	
Performance Recognition Domain	0.263 **	0.584 **	0.262 **	0.478 **	------

Note: ** *p* < 0.01. Values in bold indicate domains with statistically significant differences between the studied agencies according to the ANOVA test.

**Table 6:** Improvement recommendations.

Dimension	Immediate Action	Expected Impact
Work Demands	Shift redesign in Public Safety	Reduction in burnout
Leadership	Coaching for middle management	Improvement in work climate and retention
Recognition	Non-monetary incentive program	Increase in institutional commitment

## Data Availability

The data supporting the findings of this study are available from the corresponding author upon reasonable request. The raw data are not publicly available due to ethical and confidentiality restrictions established in the institutional agreements with the City Council of Zacatecas and the provisions of the Mexican Official Standard NOM-035-STPS-2018, intended to protect the anonymity of the participating civil servants and the sensitive nature of information regarding public safety agencies.

## Abbreviations

The following abbreviations are used in this manuscript:

DIF	National System for the Integral Development of the Family
INMUZAI	Municipal Institute for Women of Zacatecas for Equality
NOM-035-STPS-2018	Official Mexican Standard 035 of the Ministry of Labor and Social Welfare
STPS	Ministry of Labor and Social Welfare
IBM SPSS	Statistical Package for the Social Sciences
ANOVA	Analysis of Variance
*rho*	Spearman’s rank correlation coefficient
